# Morphometric analysis of feedforward pathways from the primary somatosensory area (S1) of rats

**DOI:** 10.1590/1414-431X20155115

**Published:** 2016-05-10

**Authors:** A.L. de Sá, C.P. Bahia, V.C. Correa, I.A. Dias, C. Batista, W. Gomes-Leal, A.L.S. Pinho, J.C. Houzel, C.W. Picanço-Diniz, A. Pereira

**Affiliations:** 1 Instituto do Cérebro, Universidade Federal do Rio Grande do Norte, Natal, RN, Brasil; 2 Laboratório de Neuroplasticidade, Instituto de Ciências da Saúde, Universidade Federal do Pará, Belém, PA, Brasil; 3 Laboratório de Neuroproteção e Neurorregeneração Experimental, Instituto de Ciências Biológicas, Universidade Federal do Pará, Belém, PA, Brasil; 4 Departamento de Estatística, Universidade Federal do Rio Grande do Norte, Natal, RN, Brasil; 5 Laboratório de Fronteiras em Neurociências, Instituto de Ciências Biomédicas, Universidade Federal do Rio de Janeiro, Rio de Janeiro, RJ, Brasil; 6 Laboratório de Investigações em Neurodegeneração e Infecção, Instituto de Ciências Biológicas, Universidade Federal do Pará, Belém, PA, Brasil

**Keywords:** Somatosensory cortex, Barrel field, Feedforward networks, Axon terminal, Axon morphometry

## Abstract

We used biotinylated dextran amine (BDA) to anterogradely label individual axons projecting from primary somatosensory cortex (S1) to four different cortical areas in rats. A major goal was to determine whether axon terminals in these target areas shared morphometric similarities based on the shape of individual terminal arbors and the density of two bouton types: *en passant* (Bp) and *terminaux* (Bt). Evidence from tridimensional reconstructions of isolated axon terminal fragments (n=111) did support a degree of morphological heterogeneity establishing two broad groups of axon terminals. Morphological parameters associated with the complexity of terminal arbors and the proportion of beaded Bp *vs* stalked Bt were found to differ significantly in these two groups following a discriminant function statistical analysis across axon fragments. Interestingly, both groups occurred in all four target areas, possibly consistent with a commonality of presynaptic processing of tactile information. These findings lay the ground for additional work aiming to investigate synaptic function at the single bouton level and see how this might be associated with emerging properties in postsynaptic targets.

## Introduction

Nocturnal rodents such as rats and mice rely on whisker contacts with external objects to gather information from their peri-individual space (1 [Bibr B02]). Tactile inputs from the whiskers are transduced by mechanoreceptors and make synaptic connection in the brainstem trigeminal nuclei and the thalamus before reaching the cortex (for review, see 2). At least three parallel pathways carry ascending tactile information to the cortex: the lemniscal, extralemniscal and paralemniscal pathways, which are relayed by distinct regions in the thalamus, the ventral posteromedial nucleus in the lemniscal pathway and the posterior medial nucleus in the extralemniscal and paralemniscal pathway, respectively (3,4).

The main target of thalamocortical axons is the primary somatosensory area (S1), which in rodents is arranged cytoarchitectonically in two divisions: a granular zone characterized by dense cell aggregates in layer IV called barrels, and a cell-sparse dysgranular zone comprised by septa and other regions surrounding the barrel field (3,5). Even though barrels are also present in regions representing other body parts in S1 (6), barrels associated with the whiskers are larger and have a distinct isomorphic arrangement in the posteromedial barrel subfield (PMBSF) resembling the spatial distribution of whiskers on the snout (5).

From S1, somatosensory information goes through several additional processing stages in higher-order areas. This processing is not strictly hierarchical, given that many feedback projections intervene in the process (7). From S1, information is sent simultaneously to the secondary somatosensory area (S2), the parietal ventral area (PV), the parietal rhinal area (PR), and the contralateral S1 (S1c) (8–13) where it is integrated spatiotemporally (14). Similar to S1, areas S2 and PV are also organized topographically, with a complete representation of the contralateral body, and also receive direct thalamocortical inputs (15,16). PR, on the other hand, receives projections from S2 and PV, but does not have a well-defined topographical organization. PR is located in the posterior insula and receives auditory and somatosensory inputs in rats (17).

Some studies have shown that morphological attributes of axon terminals, such as the size of terminals (18), are associated with different functional roles in neuronal pathways (19 [Bibr B20]). However, even though there is ample evidence of parallel processing within modality-specific sensory channels (e.g., 20), there are few examples of morphologically distinct types of axon terminals. For instance, glutamatergic corticocortical pathways in rodents are classified into two types, called class 1 and class 2, based on terminal morphology (21–23).

We used anterograde neuronal tracer injections to compare the morphology of feedforward axon's terminal fragments projections from S1 to higher order somatosensory areas. Our aim was to compare the morphology of these pathways and contribute to the understanding of their role in somatosensory processing. Our results suggest that information from S1 reach its targets through two parallel pathways. In a step towards classification, we present evidence for differences in the density of two types of boutons, *terminaux* and *en passant*, in feedforward projections from S1.

## Material and Methods

Male adult Wistar rats (300–350 g; n=8) were obtained from the Central Animal Facility of the Universidade Federal do Pará (UFPA), Brazil. Experimental procedures followed the Guide for the Care and Use of Laboratory Animals (NIH publication, No. 86–23, revised 1985) and were approved by the UFPA’s Ethics Committee for the Use of Animals (BIO015-09). All efforts were made to reduce the number of animals used and to avoid suffering.

One day before surgery, rats were premedicated with dexamethasone (1.0 mg/kg, *im*) to prevent brain edema and with vitamin K (1.0 mg/kg, *im*) to avoid excessive bleeding during surgery. Immediately before surgery, animals received a dose of atropine sulfate (0.1 mg/kg, *im*) and anesthesia was induced with ketamine (100 mg/kg, *im*) and xylazine (5 mg/kg, *im*). If necessary, supplementary doses of ketamine (100 mg/kg, *im*) were provided during the surgical procedure. Body temperature was maintained at about 37°C with the aid of a heating pad (Harvard Bioscience Co., USA).

### Surgical procedures and tracer injection

The head of the animal was secured in a stereotaxic apparatus (David Kopf, Germany) and a single burr hole was made at the stereotaxic coordinates AP –2.0, ML 5.0 mm, corresponding to the PMBSF in S1. Then, the dura mater was punctured and a single iontophoretic injection of 10% biotinylated dextran amine 10 KD (BDA, Molecular Probes, USA) diluted in saline phosphate buffer (PBS, pH 7.4, 0.1 M) was made through a glass capillary (20–30 µm internal tip diameter) by applying 5 µA positive current pulses (7s ON, 7s OFF) over 3–5 min using a current source (Stoelting Co, USA). We aimed for this procedure to be consistently reproducible in order to guarantee a reasonable degree of representativeness for the labeled terminals originating from that specific region. The animals were allowed to recover in their own cages with food and water *ad libitum*. After 15 days, they were anesthetized with an overdose of ketamine (24) and perfused transcardially with PBS followed by 4% paraformaldehyde in phosphate buffer (PB, pH 7.4, 0.1 M). The brains were removed from the skull and cut with a vibratome (Pelco, USA) into serial, 150-µm thick coronal sections. Sections were washed three times, 20 min each, in PB and once in a solution of 3% Triton X-100 in PB, before being incubated overnight, free-floating in the avidin/biotin/peroxidase complex (ABC, 1:200; Vector Laboratories, USA) at room temperature under constant agitation. Peroxidase labeling was revealed using the diaminobenzidine reaction intensified with nickel ammonium sulfate (25). Finally, sections were dehydrated in rising alcohol concentrations, cleared in xylene and coverslipped with Entellan (Merck, Germany). After reconstruction, the sections were processed with Nissl staining to allow delineation of cortical.

### Morphometry

For each animal, all consecutive sections were first checked for the absence of retrogradely labeled cells located distant from the immediate vicinity of the injection site. Labeled axons arising from the injection site were then examined at both low and high magnification. Individual axons were followed up to their entry into the grey matter, and individual terminal branches arborizing into target cortical areas were finally selected for computer-assisted 3D reconstruction on the basis of the following criteria: absence of branching points previous to entry in the target cortex (with the exception of the cut end of the thicker parental branch) and the entire arbor of the axon terminal should appear to be contained within a single thick section. In order to reduce sampling bias, only 1–5 terminal branches were selected per area in each animal (Table 1). After selection, well-labeled axon terminal fragments in S2 (n=25), PV (n=27), PR (n=31), and S1c (n=28) were reconstructed directly from coronal sections using a 60× oil immersion objective installed on an Optiphot-2 microscope (NIKON, Japan) equipped with a high-resolution Lucivid micromonitor (MBF Bioscience, USA) attached to a drawing tube and a 3D-motorized stage MAC5000 (Ludl, USA). After, all 3D-reconstructed sections were stained with the Nissl method to reveal the architecture of cortical layers. The devices were connected to a desktop computer running the Neurolucida software (MBF Bioscience, USA), thereby allowing for the recording and analysis of x, y, and z coordinates of digitized points. Photomicrographs were taken with a digital camera attached to the microscope; image brightness and contrast were adjusted offline with Adobe Photoshop (Adobe Systems, USA).

**Table:**
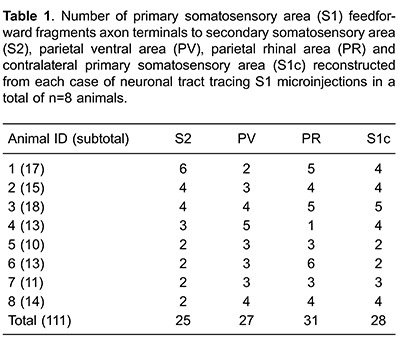

### Statistical analysis

The following morphometric parameters of axon terminal fragments were analyzed in ipsilateral S1, S2, PV, PR, and contralateral S1c: density of *en passant* boutons per millimeter (number of Bp per millimeter, Bpd), density of *terminaux* boutons per millimeter (number of Bt per millimeter, Btd), total density of boutons per millimeter (BTd: Bpd plus Btd), density of branching points (number of bifurcations per millimeter), density of segments (number of segments per millimeter), average length (total length per segment), index of Bp (number of Bp divided by the total number of boutons) and index of Bt (number of Bt divided by the total number of boutons). We did not correct for tissue shrinkage, since our study was eminently comparative and based on parameters not affected by shrinkage. To assess the homogeneity of the axonal population in each area in relation to defined morphometric variables, we first performed a multivariate analysis of variance (MANOVA). Next, an exploratory cluster analysis of morphological terminal types was performed using hierarchical clustering analysis (HCA) to explore whether specific groups of terminals existed in our sample based on the morphometric variables mentioned above (26–28). The significance of the classification performed by the HCA was tested with MANOVA. Based on the resulting classification, discriminant analysis was used to identify the variables that contributed most strongly to the separation. Average values for morphometric parameters are reported as means±SE and compared across different groups using analysis of variance (ANOVA) and the Tukey *post hoc* test, with α=0.05.

### Technical considerations

One possible methodological issue is the fact that axon terminals represent only a fragment of the parental axon arbor and it is possible that different reconstructed fragments may originate from the same parental axon. While this possibility would not affect the morphological appearance of the fragments, it could introduce some bias in the study since larger axonal arbors may have more labeled terminals and therefore a higher probability to contribute to the sample. We tried to offset this bias by reconstructing only one terminal fragment per histological section for each target area, as shown in Table 1.

## Results

**Figure 1.**
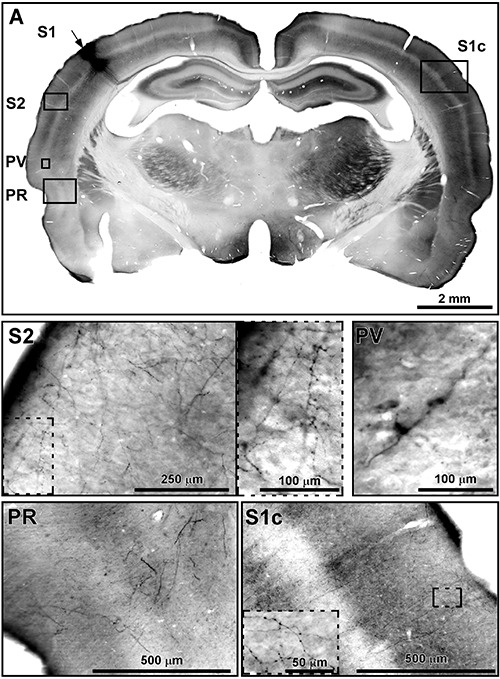
Photomicrographs of anterograde labeling of corticocortical axons after a single iontophoretic injection of BDA in S1. *A*, Low-power photomontage (×20 objective virtual slide) of an entire coronal section showing the location of a typical injection site in area S1 (arrow) and of target areas containing anterogradely labeled axons. Below, photomicrographs with variable magnifications illustrating anterogradely-labeled axons originating in S1 and terminating within the second somatosensory area (S2), the parietal-ventral area (PV), the parietal-rhinal area (PR), and the contralateral S1 (S1c). Insets show branches with boutons *terminaux* and *en passant*. Scale bars are indicated for each panel.

**Figure 2.**
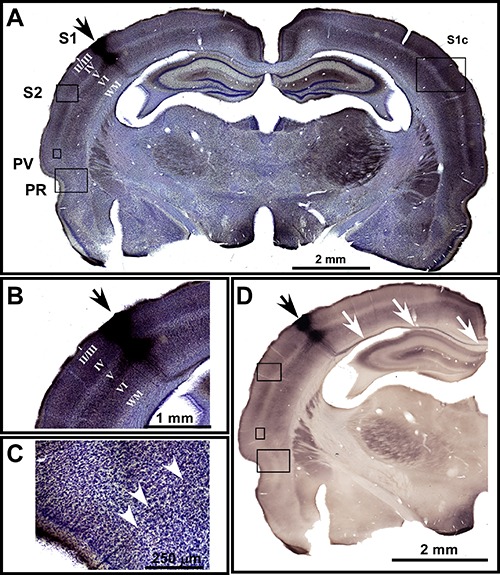
Histological rat brain section double-stained with Nissl showing the laminar location of labeled corticocortical axon terminals after a single iontophoretic injection of biotinylated dextran amine in S1. *A*, Low-power photomontage of the same coronal section depicted in Figure 1. *B*, Photomicrograph showing cortical layers and *C*, anterogradely-labeled axon terminals in a section double-stained with Nissl. Arrows indicate axon terminal segments. *D*, Black arrow indicates injection site and white arrows indicate callosal axons. The boxes indicate cortical areas (S2, PV and PR) where axon fragments labeled with DBA were reconstructed. Cortical layers are indicated by roman numerals, from I to VI; WM: white matter.

Morphological analysis was based on a sample of 111 BDA-labeled terminal fragments (see Table 1). All BDA iontophoretic injections were confined to S1 and exhibited a dense black central core, ranging from 300–500 µm in diameter, surrounded by anterogradely-labeled cell bodies and axonal fragments belonging to intracortical circuits and spanning layers II to VI (Figure 1). Cortical layers could be discerned on sections counterstained with Nissl (Figure 2) due to the faint background staining from the diffuse peroxidase activity under lower magnification (Figure 1). Only layer I was not shown because it was poorly labeled and couldn’t be well discerned as can be seen in Figure 2A and B. We did not find retrogradely labeled cell bodies outside the immediate vicinity of the iontophoretic injection sites. Labeled axons could be followed from S1 to target regions located in ipsilateral S2, PV, and PR areas. Callosal axon terminals were also found in homotopic regions in the contralateral S1. Terminal axon segments bearing boutons were located in all cortical layers, except layer I (Figures 1 and 2). This is in accordance with their feedforward nature (29). We could identify both Bp and Bt boutons studding from axon fragments labeled with BDA. The former can be associated with a thickening of the axon, while the latter resemble spiny appendages located at the endings of axonal branches.

The average morphometric parameter values for axon fragments located in S1 targets were as follows: segment length per millimeter (S2=0.12±0.01; PV=0.15±0.02; PR=0.12±0.01; S1c 0.16±0.01), segment density per millimeter (S2=10.47±1.0; PV=9.31±1.3; PR=9.66±0.6; S1c=9.06±0.9), number of branching points per millimeter (S2=5.64±0.50; PV=5.41±0.53; PR=5.77±0.62; S1c=5.23±0.59), and total bouton density per millimeter (Bp plus Bt; S2=67.54±7.9; PV=60.00±8.7; PR=49.51±5.8; S1c=65.18±7.4).

MANOVA analysis did not demonstrate any significant segregation of morphometric variables, according to target area. Thus, axon fragments located in S2, PV, PR, and S1c seem to comprise a homogeneous population characterized by strong morphological similarities (Wilks test: F=1.1122, P=0.3393; Hotelling-Lawley test: F=1.1145, P=0.3367).

**Figure 3.**
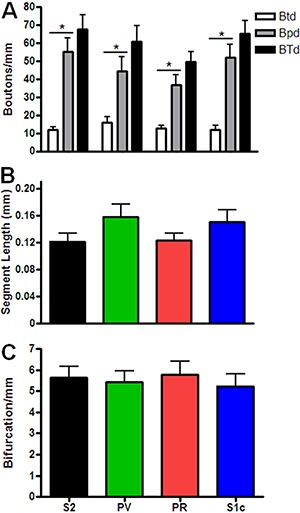
Multivariate discriminant statistical analysis shows that the linear density per millimeter of *en passant* boutons (Bpd) was higher than that of *terminaux* boutons (Btd) (*P<0.05, Bpd *vs* Btd; ANOVA-Tukey *post hoc* test) in all cortical target areas (*A*). BTd: Bpd+ Btd. Axon terminals in secondary somatosensory area (S2), parietal rhinal area (PR), parietal ventral area (PV) and contralateral primary somatosensory area (S1c) presented similar morphological components (P>0.05) based on segment length per millimeter (*B*) or bifurcation density per millimeter (*C*).

ANOVA demonstrated significant differences (F=1.2; P≤0.05) in the relative number of Bp and Bt within individual cortical areas (Figure 3). Interestingly, the total density of boutons appeared to be similar in all target areas (Figure 3B). This may suggest that the synaptic efficacy of these pathways is similar in those areas. This is different from intracortical circuit connections, for instance, where connectivity is a function of spatial separation between neurons (30).

**Figure 4.**
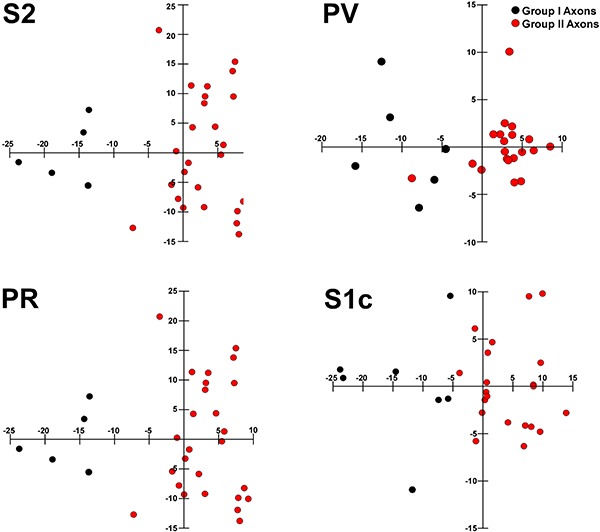
Graphic results of discriminant analysis showing the separation of axon terminals from secondary somatosensory area (S2), parietal rhinal area (PR), parietal ventral area (PV) and contralateral primary somatosensory area (S1c) into two distinct groups: Group I axons (black dots) and Group II Axons (red dots).

The discriminant analysis confirmed the separation of the data in two morphologically distinct groups and also revealed which variables were most important for classification (Figure 4). The discriminant analysis produced two linear functions having weight coefficients for each morphometric variable. The equations for S2 are as follows: Y1=-0.1195×X1 -0.1352×X2 -0.3881×X3 +0.9038×X4 and Y2=0.1630×X1 -0.0036×X2 -0.1392×X3 + 0.9768×X4. It is worth mentioning that dimension Y1 is already sufficient to separate terminal groups in S2, as can be seen in Figure 4, where group I is associated with values smaller than group II. The same pattern is replicated for the remaining targets: PV (Y1=-0.0245×X1 -0.1637×X2 -0.4955×X3 + 0.8527×X4 and Y2=0.0163×X1 -0.0572×X2 + 0.2295×X3 + 0.9715×X4), PR (Y1=-0.0104×X1 -0.2576×X2 -0.3650×X3 + 0.8946×X4 and Y2=0.9666×X1 + 0.0257×X2 -0.0471×X3 + 0.2476×X4), and S1c (Y1=-0.2408×X1 -0.2290×X2 -0.5083×X3 + 0.7945×X4 and Y2=-0.2498×X1 -0.0089×X2 + 0.3284×X3 + 0.9083×X4).

**Figure 5.**
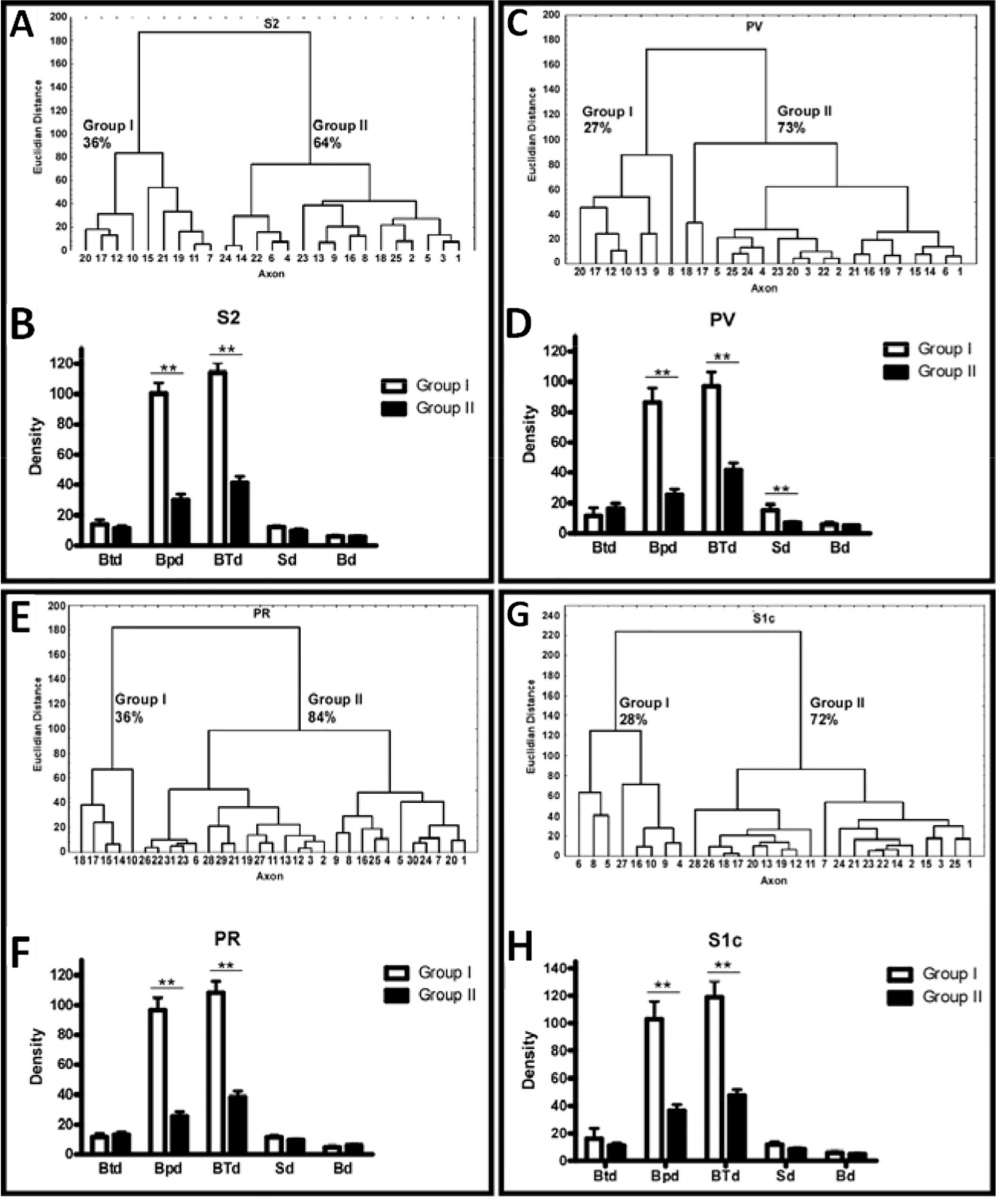
Hierarchical cluster analysis (HCA) dendrogram showing that feedforward axon terminals from S1 can be separated into two groups (I and II) in secondary somatosensory area (S2) (*A*, *B*), parietal ventral area (PV) (*C, D*), parietal rhinal area (PR) (*E*, *F*) and contralateral primary somatosensory area (S1c) (*G*, *H*). The variable that most contributed to the distinction between groups was the density of boutons *terminaux* (Btd) (*P<0.05, ANOVA-Tukey *post hoc* test). Sd: segment density per millimeter; Bpd: *en passant* boutons; Bd: bifurcation density per millimeter; BTd: Bpd+ Btd

Figure 5 shows the dendograms obtained with the HCA performed on data from terminals located in the four target areas. The terminals from each area are identified at the bottom of the graphs and merge into discrete clusters at distinct stages, depending on their degree of morphological similarity. The dendograms suggested the existence of two morphologically distinct groups of terminals (group I and group II) in each one of the studied areas (Figure 5) (ANOVA F=1.0; P<0.01).

**Figure 6.**
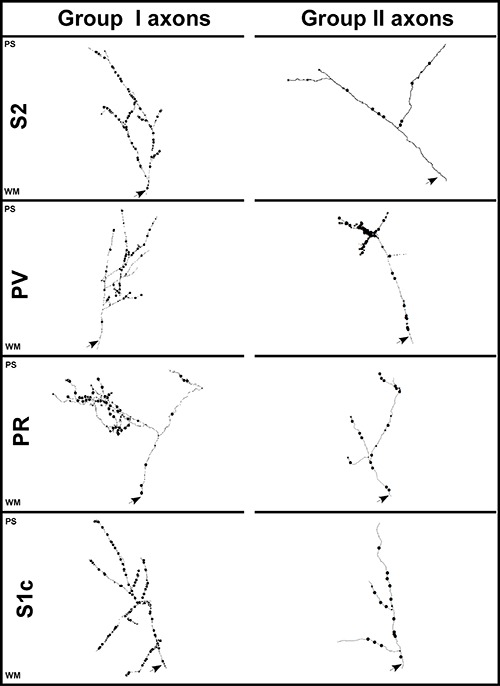
Representative examples of digitally reconstructed axon terminals in secondary somatosensory area (S2), parietal rhinal area (PR), parietal ventral area (PV) and contralateral primary somatosensory area (S1c). Terminals are separated according to their profile into groups I and II. The relative position of both the pia mater and white matter borders are depicted in the figure. Arrows indicate the parent axon branch. PS: pial surface; WM: white matter.

Axon fragments from group I displayed a higher density of Bp (S2=100±21, PV=86±9, PR=96±8 and S1c=102±1.20) and Bt (S2=13±9, PV=11±5, PR=11±2 and S1c=15±7) than group II fragments - Bp (S2=30±16, PV=25±3, PR=25±3 and S1c=36±4) and Bt (S2=11±7, PV=16±3, PR=13±1 and S1c=11±1), (ANOVA F=1.0; P<0.01; Figure 6).

## Discussion

The morphometric analysis of axon terminal arbors filled with BDA revealed that corticocortical projections originating in S1 and targeting somatosensory areas in both hemispheres seem to be morphologically similar. According to our results, despite this similarity, intrinsically associated with the density of two types of presynaptic boutons (beaded *en passant* boutons and stalked *terminaux* boutons) and the geometry of terminal arbors, the HCA suggested the presence of more than one group of terminals. This was further confirmed by MANOVA and by discriminant analysis (Figures 5 and 6).

Regarding the differences on the relative density of Bt and Bp between the two terminal groups, the question is whether there is any functional correlation associated with this finding. Both types of boutons have been structurally associated with synapses (31,32). Even though the precise relationship between form and function in this case is still not determined, it has been proposed that Bt could be more involved with presynaptic facilitation and show more structural plasticity than Bp, due to more efficient calcium storage (33). The findings from De Paola (34) suggest the possibility of a difference in the plastic potential between the two terminal groups we found in this work.

In rodents, tactile information from the whiskers is conveyed to S1 by at least 3 pathways (3,4,34,35). These pathways have been implicated with carrying information about distinct whisking attributes and remain relatively segregated in S1 (35). However, very little is known about their relative contribution to feedforward projections from S1 to S2, PV, PR, S1c. In the cortex, based on studies on synaptic properties and anatomical features, Sherman and Guillery (23) reported that glutamatergic projections can be classified into Class 1 and Class 2, depending on their role as circuit drivers or modulators, respectively. This separation between the driving and modulatory functions of glutamatergic projections can also be seen in the somatosensory pathways mentioned above and that carry information from the whiskers to S1, through synaptic relays in the trigeminal nuclei to the thalamus (16). The study by Viaene and coworkers (16) suggests that the role of the paralemniscal pathway is to provide modulatory inputs to S1, while the lemniscal pathway conveys precise information about whisker deflections to S1 and plays a role in object localization and identification (35). The modulatory role of the paralemniscal pathway (16) is also under the influence of the locus coeruleus (36). The most conspicuous morphological difference between drive and modulator pathways lies on the size and shape of boutons, with smaller Bt associated with driving connections and larger ones associated with a modulatory role (37).

While the size of Bp might affect axon dynamics, it is reasonable to suppose that such dynamics can also be affected later by other structural aspects of axon terminals, such as the relative distribution of Bp and Bt (33). As discussed above, Bt can probably facilitate synaptic potentials (33) in an activity-dependent manner (38). Such dynamic control of synaptic sensitivity could increase both the sensitivity and fidelity of transmission of sensory signals along driving pathways. Bp, on the other hand, could have a more modulatory effect, extending the functional reach of lemniscal and paralemniscal pathways beyond S1. The differences in the profile of group I and II terminals can also affect their susceptibility to plasticity, as evidenced by studies in the adult visual cortex of both rodents and primates (39,40) showing that the turnover rate of Bt is significantly higher than Bp (40).

Our results suggest that feedforward projections from S1 are sent to at least 4 other cortical regions, including contralateral S1, in the rat. Morphologically, these inputs are very similar and can be further subdivided into two classes of terminals. Other studies had previously presented evidence for the existence of two categories of glutamatergic terminals in corticocortical pathways in the visual cortex. In the visual cortex, these two groups of terminals can be differentiated in terms of the functional role they play in their targets (driving or modulatory). In the somatosensory cortex, they could provide a substrate for the continuing segregation of parallel pathways beyond S1.
